# Temporal rise in the proportion of younger adults and older adolescents among coronavirus disease (COVID-19) cases following the introduction of physical distancing measures, Germany, March to April 2020

**DOI:** 10.2807/1560-7917.ES.2020.25.17.2000596

**Published:** 2020-04-30

**Authors:** Edward Goldstein, Marc Lipsitch

**Affiliations:** 1 Center for Communicable Disease Dynamics, Department of Epidemiology, Harvard T.H. Chan School of Public Health, Boston, United States; 2 Department of Immunology and Infectious Diseases, Harvard T.H. Chan School of Public Health, Boston, United States

**Keywords:** COVID-19, age groups, younger adults, older adolescents

## Abstract

Using data on coronavirus disease (COVID-19) cases in Germany from the Robert Koch Institute, we found a relative increase with time in the prevalence in 15–34 year-olds (particularly 20–24-year-olds) compared with 35–49- and 10–14-year-olds (we excluded older and younger ages because of different healthcare seeking behaviour). This suggests an elevated role for that age group in propagating the epidemic following the introduction of physical distancing measures.

The ongoing pandemic of severe acute respiratory syndrome coronavirus 2 (SARS-CoV-2) has caused more than 1,991,000 detected cases of coronavirus disease (COVID-19) illness worldwide and claimed more than 130,800 lives as at 16 April 2020 [1]. As rates of contact between individuals in different age groups under the current physical distancing measures are expected to depart considerably from the regular mixing patterns [2], there is uncertainty regarding the role of different age groups in propagating the SARS-CoV-2 epidemics in different countries. While disease is most severe in older age groups, a sizeable share of COVID-19-related hospitalisations in the United States and other countries occurs in individuals aged 20–55 years [3]. A study of close contacts of COVID-19 cases in China found comparable rates of infection with SARS-CoV-2 in different age groups [4]. South Korea employed one of the most inclusive testing practices for SARS-CoV-2 in the world, and the rate of detected cases in South Korea is highest in persons aged 20–29 years [5]. 

Here, we apply a methodology we developed earlier [6,7] to data from the Robert Koch Institute on COVID-19 cases in Germany [8] to assess the relative roles of different age groups in SARS-CoV-2 transmission. 

## Role of different age groups during the epidemic

The data from the Robert Koch Institute represent a national database [8] of cases reported under the German ‘Act on the Prevention and Control of Infectious Diseases in Man’ [9]. We expect that groups that have an elevated role in propagating an epidemic will increase their share among all incident cases during the early stages of the epidemic. For example, if contact rates for one age group are less affected by physical distancing measures than for another age group because of lesser adherence, the incidence of infection in the former age group will increase relative to the latter after the physical distancing measures are implemented. In addition, if a country has regions with different growth rates for their epidemics, the age group that drives the incidence of infection in regions with faster growing and larger epidemics will increase its share among all incident cases in the country. 

Temporal increase in the share of a given age group among all cases of infection can be evaluated using the relative risk (RR) statistic that estimates the ratio of the proportion of a given age group among all detected (reported) cases of COVID-19 for a later time period vs an early time period. We selected the early period to be weeks 10–11, 2020 (shortly before the introduction of physical distancing measures), and the later period to be weeks 13–14, 2020. In Germany, the initial set of physical distancing measures, including the closure of non-essential businesses and sports and entertainment venues, was introduced on 16 March (beginning of week 12), while further lockdown measures, including school closures and the ban on public gatherings of more than two persons, were introduced on 22 March (end of week 12). Note that at the time of data access (15 April 2020), data for week 14, 2020 were incomplete. 

We included eight 5-year age groups in our analysis, from 10–14 years through 45–49 years (see Discussion regarding the exclusion of other age groups). For each age group *g*, let *E(g)* be the number of detected COVID-19 cases in age group *g* during the early period, and *L(g)* be the corresponding number during the later period. The RR statistic ([6,7]) is

$$RR\left(g\right)=\frac{L(g)}{\sum_{h=1}^{8}L(h)}/\frac{E(g)}{\sum_{h=1}^{8}E(h)}\;(Formula\;1)$$

The logarithm ln(*RR*(*g*)) of the RR in the age group *g* is approximately normally distributed [10], with the standard error

$$SE=\sqrt{\frac{1}{L(g)}+\frac{1}{E(g)}-(\frac{1}{\sum_{h=1}^{8}L(h)}+\frac{1}{\sum_{h=1}^{8}E(h)})}\;(Formula\;2)$$

For each pair of age groups *g*1 and *g*2, the relative risks *RR*(*g*1) and *RR*(*g*2) are compared using the odds ratio (OR)

OR(g1,g2) = RR(g1)/RR(g2) (Formula 3)

It follows from Formula 1 that *OR*(*g*1,*g*2) equals 

$$OR=\;\frac{L(g1)}{E(g1)}/\frac{L(g2)}{E(g2)}\;\;(Formula\;4)$$

which is the OR for a COVID-19 case to be in age group *g*1 vs *g*2 for the later vs early period. Estimates for pairwise OR were performed using Fisher’s exact test.

### Ethical statement

We used publicly available data on COVID-19 cases in Germany from the Robert Koch Institute (https://survstat.rki.de), with no informed consent sought [8].

## Results

Table 1 shows, for the eight age groups included in our study, the weekly number of reported COVID-19 cases in Germany [8] for weeks 10–14, 2020.

**Table 1:** *. Weekly number of reported COVID-19 cases in the eight age groups included in the study, Germany, weeks 10–14, 2020 (n = 35,726)

Age group (years)	Week 10	Week 11	Week 12	Week 13	Week 14
10–14	21	97	234	359	111
15–19	40	163	509	897	287
20–24	70	304	1,356	2,094	585
25–29	70	510	1,889	2,489	648
30–34	69	497	1,941	2,436	656
35–39	59	523	1,668	2,202	610
40–44	80	525	1,715	2,158	633
45–49	97	701	1,979	2,655	715

COVID-19: coronavirus disease.

Note that data for week 14 were incomplete as at 15 April 2020 [8].

Table 2 shows the estimates for *RR*(*g*) for different age groups for the later period (weeks 13–14) vs the early period (weeks 10–11). The highest RR was estimated for individuals aged 20–24 years, followed by individuals aged 15–19, 30–34 and 25–29 years. Estimates of the RR for individuals older than 35 years and those aged 10–14 years were lower.

**Table 2:** Relative risks for COVID-19 cases of being in a given age group during the later period (weeks 13–14) vs early period (weeks 10–11) for each of the eight age groups included, Germany, weeks 10–14, 2020 (n = 35,726)

Age group (years)	Relative risk	95% CI
10–14	0.78	0.64–0.95
15–19	1.14	0.99–1.32
20–24	1.4	1.27–1.55
25–29	1.06	0.98–1.15
30–34	1.07	0.99–1.16
35–39	0.95	0.87–1.03
40–44	0.9	0.83–0.98
45–49	0.83	0.77–0.89

CI: confidence interval; COVID-19: coronavirus disease.

Note that the data for week 14 were incomplete when extracted from [8] on 15 April 2020.

Table 3 shows the estimated OR for being a detected COVID-19 case during the later vs early period for one age groups vs the other (Formula 3). For the age group 20–24 years, the OR relative to any other age group for being a case during the later vs the early period was significantly above 1. For the age group 15–19 years, this OR was significantly above 1 relative to any of the age groups 35–39, 40–44, 45–49 and 10–14 years. For the age groups 25–29 and 30–34 years, this OR was significantly above 1 relative to any of the age groups 40–44 , 45–49 and 10–14 years. 

**Table 3:** Odds ratios for different pairs of age groups, for a COVID-19 case to occur in the later period (weeks 13–14) vs early period (weeks 10–11), Germany, weeks 10–14, 2020 (n = 35,726)

Age group (years)	15–19	20–24	25–29	30–34	35–39	40–44	45–49
10–14	**0.68** **(0.53–0.89)**	**0.56** **(0.44–0.71)**	**0.74** **(0.59–0.93)**	**0.73** **(0.58–0.92)**	0.82 (0.66–1.04)	0.86 (0.69–1.09)	0.94 (0.76–1.18)
15–19		**0.81** **(0.68–0.98)**	1.08 (0.9–1.29)	1.07 (0.9–1.28)	**1.21** **(1.01–1.44)**	**1.26** **(1.06–1.51)**	**1.38** **(1.17–1.64)**
20–24			**1.32** **(1.15–1.53)**	**1.31** **(1.14–1.51)**	**1.48** **(1.29–1.71)**	**1.55** **(1.35–1.79)**	**1.7** **(1.48–1.94)**
25–29				0.99 (0.87–1.13)	1.12 (0.99–1.27)	**1.17** **(1.03–1.33)**	**1.28** **(1.14–1.44)**
30–34					1.13 (0.99–1.29)	**1.18** **(1.04–1.35)**	**1.29** **(1.15–1.46)**
35–39						1.05 (0.92–1.19)	**1.14** **(1.02–1.29)**
40–44							1.09 (0.97–1.23)

COVID-19: coronavirus disease.

Each entry represents an estimate of the odds ratio for the age group in the corresponding row vs the age group in the corresponding column. 95% confidence intervals are shown in brackets.

Note that the data for week 14 were incomplete when extracted from [8] on 15 April 2020.

## Discussion

There is a great deal of uncertainty about the role of different age groups in propagating the ongoing COVID-19 epidemics in different countries. Some evidence about those relative roles can be obtained by examining temporal changes in the proportions of different age groups among detected COVID-19 cases. Our results, based on applying the RR statistic [6,7] to data on COVID-19 cases in Germany [8] suggest that individuals aged 15–34 years (particularly the 20–24-year-olds) had their share in SARS-CoV-2 incidence increase with time compared with the 35–49-year-olds and children aged 10–14 years. Further work is needed to assess the reasons behind those relative increases, including the possibility of elevated mixing because of lesser adherence to physical distancing guidelines for persons aged 15–34 years. 

Our study has some limitations. Data published for week 14 were incomplete at the time when the analysis was performed. Having the later period to be week 13 alone rather than weeks 13-14 has a very minor effect on the results of the paper (Supplementary material). We assess relative changes in the incidence of SARS-CoV-2 cases in different age groups in Germany using data on detected (reported) COVID-19 cases [8]. The ratio between the number of detected COVID-19 cases and incident cases of SARS-CoV-2 infection (case detection rate) in each age group may vary with time because of changes in healthcare-seeking behaviour and other factors. We believe that it is unlikely that the likelihood of seeking medical care/being tested increased substantially more for 15–34-year-olds than for 35–49- or 10–14-year-olds as the risk of severe disease is generally smaller in the former age groups compared to the latter. In addition, to address the possibility that testing practices during the early period focused on specific age or risk groups, we excluded week 9 from the early period and conducted sensitivity analyses (Supplementary material) with the early period assumed to be week 11 only (rather than weeks 10–11). In those sensitivity analyses, there was a further increase in RR for the 15–24 year age group, and the RR estimates in the 25–34 year age group remained higher than in age groups 35–49 and 10–14 years. All of this suggests that the observed temporal increases in the relative share of individuals aged 15–34 years among detected COVID-19 cases aged 10–49 years should be indicative of an actual increase in the prevalence of 15–34-year-olds among SARS-CoV-2 infections in the German population aged 10–49 years. In addition, data on mixing patterns in different age groups during regular times [2] suggest that those age groups are expected to have the largest role in driving the current SARS-CoV-2 pandemic Moreover, the age distribution of detected cases in South Korea [5] further supports our conclusion about the role of younger adults during SARS-CoV-2 epidemics. Finally, because it is possible that symptomatic older adults and younger children may be more likely to seek medical care/get tested compared with other age groups as the awareness about the epidemic increases with time, those age groups were not included in our analyses based on the RR statistic. 

We believe that despite those limitations, our results provide evidence about the growing role of younger adults (particularly those aged 20–24 years) and older adolescents following the introduction of physical distancing measures for the 2020 COVID-19 epidemic in Germany. Those results may be relevant to informing physical distancing efforts, particularly for younger adults and older adolescents.

## *Authors’ correction

In the original article, published on 30 April 2020, the numbers in Table 1 were wrong. The Table has been corrected on request of the authors on 4 May 2020.

## Supplementary Data

26000 Supplement
