# Authors’ correction for Euro Surveill. 2020;25(17)

**DOI:** 10.2807/1560-7917.ES.2020.25.18.20200507c

**Published:** 2020-05-07

**Authors:** Eurosurveillance editorial team

**Affiliations:** 1 European Centre for Disease Prevention and Control (ECDC), Stockholm, Sweden

In the article ‘Temporal rise in the proportion of younger adults and older adolescents among coronavirus disease (COVID-19) cases following the introduction of physical distancing measures, Germany, March to April 2020’ by Goldstein and Lipsitch, published on 30 April 2020, the numbers in Table 1 were wrong. The Table has been corrected on request of the authors on 4 May 2020.

