# Supplementary material for: Temporal rise in the proportion of younger adults and older adolescents among coronavirus disease (COVID-19) cases following the introduction of physical distancing measures, Germany, March to April 2020
Source: Euro Surveill. 2020 Apr 30;25(17):2000596. doi: 10.2807/1560-7917.ES.2020.25.17.2000596 (PMC7201953; doi:10.2807/1560-7917.ES.2020.25.17.2000596)
Supplement: Supplement [file 20-00596_GOLDSTEIN_Supplement.pdf]

Supplementary Material

This supplementary material is hosted by Eurosurveillance as supporting information alongside the article *Temporal rise in the proportion of younger adults and older adolescents among coronavirus disease (COVID-19) cases following the introduction of physical distancing measures, Germany, March to April 2020?*, on behalf of the authors, who remain responsible for the accuracy and appropriateness of the content. The same standards for ethics, copyright, attributions and permissions as for the article apply. Supplements are not edited by Eurosurveillance and the journal is not responsible for the maintenance of any links or email addresses provided therein.

1. Relative risks for a different early period

Estimates of the relative risk may depend on the choice of the early period, particularly when testing practices related to certain age and risk groups (e.g. people returning from a skiing vacation) were taking shape. To mitigate the effect of testing practices during the earliest stages of the epidemic, we have have (a) excluded week 9 from the early period; (b) conducted sensitivity analyses with the early period assumed to be week 11 only (rather than weeks 10-11). The results of those sensitivity analyses are presented here.

Table S1 shows the estimates for the relative risk $RR(g)$ for different age groups for the later period (weeks 13-14, 2020) vs. early period (week 11, 2020). The estimates for the age groups 15-24y increased further compared to the analysis in the main body of the text. Similarly to the results of the analysis in the main body of the text, the highest estimate for the relative risk belongs to individuals aged 20-24y, followed by individuals aged 15-19y, 30-34y and 25-29y, with estimates of the relative risk for individuals aged over 35y and those aged 10-14y being lower.

Table S1. Relative risks for COVID-19 cases of being in a given age group during the later period (weeks 13–14) vs early period (week 11) for each of the eight age groups included, Germany, weeks 11–14, 2020 (n=34,146)

Age group	RR (relative risk)	95% CI
10-14y	0.82	(0.66,1.02)
15-19y	1.23	(1.05,1.45)

20-24y	1.50	(1.34,1.68)
25-29y	1.05	(0.96,1.14)
30-34y	1.06	(0.97,1.15)
35-39y	0.91	(0.84,1)
40-44y	0.9	(0.83,0.98)
45-49y	0.82	(0.76,0.88)

CI: Confidence Interval; COVID-19: coronavirus disease.

Note that the data for week 14 were incomplete when extracted from [1] on 15 April 2020.

Table S2 shows, for different pairs of age groups, the estimated odds ratios for being a detected COVID-19 case during the later period (weeks 13-14) vs. early period (week 11) for one age groups vs. the other. The results are similar to the results in the main body of the text except that some of the estimates in Table S2 fail to reach statistical significance compared to the corresponding estimates in Table 3, presumably due to lower counts for the early period. On the other hand, the odds ratios for age groups 25-34y vs. 35-39y are significantly above 1 in the new analysis (compare to Table 3). For the age group 20-24y, the odds ratio relative to any other age group except for persons aged 15-19y for a case to be during the later vs. early period was significantly above 1. For the age group 15-19y, the odds ratio relative to any of the following age groups: 35-39y, 40-44y, 45-49y, 10-14y for a case to be during the later vs. early period was significantly above 1. For the age groups 25-29y and 30-34y, the odds ratio relative to any of the following age groups: 35-39y, 40-44y, 45-49y for a case to be during the later vs. early period was significantly above 1.

TABLE S2. Odds ratios for different pairs of age groups, for a COVID-19 case to occur in the later period (weeks 13–14) vs early period (week 11), Germany, weeks 11–14, 2020 (n=34,146)

Age group	15-19y	20-24y	25-29y	30-34y	35-39y	40-44y	45-49y
10-14y	**0.67 (0.5,0.89)**	**0.55 (0.43,0.71)**	0.79 (0.62,1.01)	0.78 (0.61,1)	0.9 (0.71,1.16)	0.91 (0.72,1.17)	1.01 (0.8,1.29)
15-19y		0.82 (0.67,1.02)	1.18 (0.98,1.44)	1.17 (0.96,1.42)	**1.35 (1.12,1.64)**	**1.37 (1.13,1.66)**	**1.51 (1.26,1.83)**
20-24y			**1.43 (1.23,1.67)**	**1.42 (1.21,1.65)**	**1.64 (1.41,1.91)**	**1.66 (1.42,1.93)**	**1.83 (1.58,2.12)**

25-29y				0.99 (0.86,1.13)	**1.14 (1,1.31)**	**1.16 (1.01,1.32)**	**1.28 (1.13,1.45)**
30-34y					**1.16 (1.01,1.32)**	**1.17 (1.02,1.34)**	**1.29 (1.14,1.47)**
35-39y						1.01 (0.88,1.16)	1.12 (0.99,1.27)
40-44y							1.11 (0.98,1.25)

COVID-19: coronavirus disease.

Each entry represents an estimate of the odds ratio for the age group in the corresponding row vs the age group in the corresponding column. 95% confidence intervals are shown in brackets.

Note that the data for week 14 were incomplete when extracted from [1] on 15 April 2020.

2. Relative risks for a different later period

As data for week 14 are incomplete as of Apr. 26, 2020, we performed sensitivity analysis assuming that the late period represents week 13 alone (rather than weeks 13-14). Table S3 shows the estimates for the relative risk $RR(g)$ for different age groups for the later period (week 13, 2020) vs. early period (weeks 10-11, 2020). The estimates of the relative risk are very similar to the estimates in the main body of the text (Table 2), with the estimate for individuals aged 15-19y decreasing slightly. Similarly to the results of the analysis in the main body of the text, the highest estimate for the relative risk belongs to individuals aged 20-24y, followed by individuals aged 15-19y, 30-34y and 25-29y, with estimates of the relative risk for individuals aged over 35y and those aged 10-14y being lower.

Table S3. Relative risks for COVID-19 cases of being in a given age group during the later period (week 13) vs early period (weeks 10-11) for each of the eight age groups included, Germany, weeks 10–13, 2020 (n=30,407)

Age group	RR (relative risk)	95% CI
10-14y	0.76	(0.62,0.93)
15-19y	1.11	(0.95,1.28)
20-24y	1.4	(1.26,1.55)
25-29y	1.07	(0.99,1.17)

30-34y	1.08	(0.99,1.17)
35-39y	0.95	(0.87,1.03)
40-44y	0.89	(0.82,0.97)
45-49y	0.83	(0.78,0.89)

CI: Confidence Interval; COVID-19: coronavirus disease.

Table S4 shows, for different pairs of age groups, the estimated odds ratios for being a detected COVID-19 case during the later period (week 13) vs. early period (weeks 10-11) for one age groups vs. the other. The results are similar to the results in the main body of the text (Table 3) except that the odds ratio for persons aged 15-19y vs. persons aged 35-39y fails to reach statistical significance. For the age group 20-24y, the odds ratio relative to any other age group for a case to be during the later vs. early period was significantly above 1. For the age group 15-19y, the odds ratio relative to any of the following age groups: 40-44y, 45-49y, 10-14y for a case to be during the later vs. early period was significantly above 1. For the age groups 25-29y and 30-34y, the odds ratio relative to any of the following age groups: 40-44y, 45-49y for a case to be during the later vs. early period was significantly above 1.

TABLE S4. Odds ratios for different pairs of age groups, for a COVID-19 case to occur in the later period (week 13) vs early period (weeks 10-11), Germany, weeks 10–13, 2020 (n=30,407)

Age group	15-19y	20-24y	25-29y	30-34y	35-39y	40-44y	45-49y
10-14y	**0.69 (0.53,0.9)**	**0.54 (0.43,0.69)**	**0.71 (0.56,0.9)**	**0.71 (0.56,0.9)**	0.8 (0.64,1.02)	0.85 (0.68,1.08)	0.91 (0.73,1.15)
15-19y		**0.79 (0.65,0.96)**	1.03 (0.86,1.24)	1.03 (0.86,1.23)	1.17 (0.98,1.4)	**1.24 (1.03,1.49)**	**1.33 (1.12,1.59)**
20-24y			**1.3 (1.13,1.51)**	**1.3 (1.12,1.51)**	**1.48 (1.28,1.71)**	**1.57 (1.36,1.81)**	**1.68 (1.47,1.93)**
25-29y				1 (0.87,1.14)	1.13 (1,1.29)	**1.2 (1.06,1.37)**	**1.29 (1.14,1.46)**
30-34y					1.14 (1,1.3)	**1.21 (1.06,1.37)**	**1.29 (1.14,1.46)**
35-39y						1.06 (0.93,1.21)	**1.14 (1.01,1.29)**

40-44y							1.07 (0.95,1.21)

COVID-19: coronavirus disease.

Each entry represents an estimate of the odds ratio for the age group in the corresponding row vs the age group in the corresponding column. 95% confidence intervals are shown in brackets.

References

[1] Robert Koch Institute (RKI). SurvStat@RKI 2.0. Berlin: RKI. [Accessed: 4 Apr, 2020]. Available from: https://survstat.rki.de
